# Salvage endoscopic wide-field full-thickness resection of T2 rectal cancer with endoscopic submucosal dissection instruments, without defect closure

**DOI:** 10.1055/a-2081-9081

**Published:** 2023-06-07

**Authors:** Georgios Mavrogenis, Dimitrios Ntourakis, Neoklis Kritikos, Panagiotis Kasapidis, Loukas Kaklamanis, Vassilis Kouloulias, Fateh Bazerbachi

**Affiliations:** 1 Unit of Hybrid Interventional Endoscopy, Department of Gastroenterology, Mediterraneo Hospital, Athens, Greece; 2 School of Medicine, European University Cyprus, Nicosia, Cyprus; 3 Department of Surgery, Mediterraneo Hospital, Athens, Greece; 4 Department of Pathology, Mediterraneo Hospital, Athens, Greece; 5 Department of Radiotherapy, Mediterraneo Hospital, Athens, Greece; 6 CentraCare, Interventional Endoscopy Program, St. Cloud Hospital, St. Cloud, Minnesota, USA

A 86-year-old woman was referred for recurrent bleeding secondary to a 4-cm cT2N0 cancer of the distal rectum ( Fig. 1 ). Tumor staging was negative for metastasis. She had a history of severe aortic stenosis and atrial fibrillation under anticoagulants, making her a poor candidate for surgical treatment by total mesorectal excision. After a multidisciplinary approach and informed patient consent, we performed a palliative endoscopic resection of the tumor by means of endoscopic submucosal techniques under propofol sedation (Video 1 ).

**Fig. 1:**
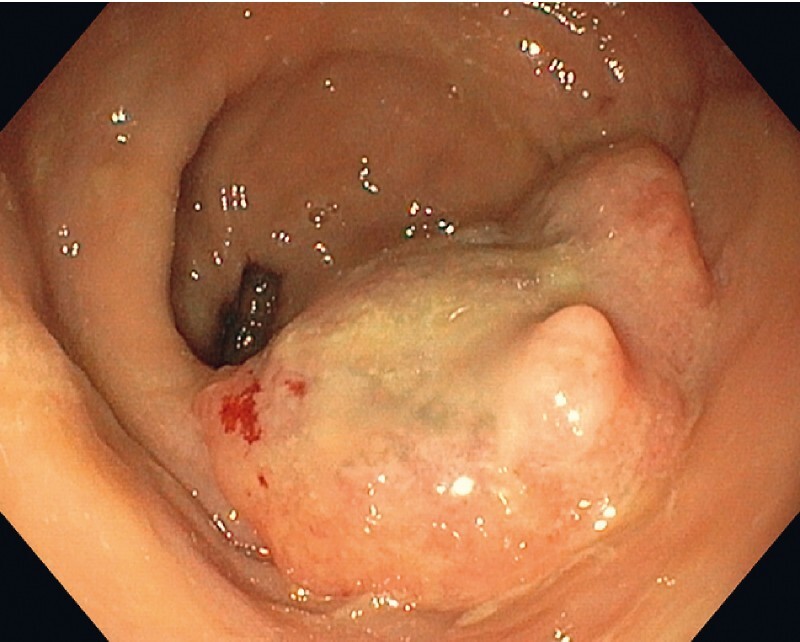
A 4-cm tumor of the lower rectum.

**Video 1** Salvage endoscopic wide-field full-thickness resection of T2 rectal cancer with endoscopic submucosal dissection instruments, without defect closure.


After circumferential muscular incision ( Fig. 2 , Fig. 3 ), the specimen was progressively dissected from the perirectal fat using a square tip endoscopic submucosal dissection (ESD) knife (Square-Knife, Endoaccess, Garbsen, Germany) in spray coagulation mode (Effect 3.0, VIO3, ERBE, Tübingen, Germany). Large perirectal vessels were coagulated with hot biopsy forceps. No major bleeding was encountered. At the end of the procedure, which lasted 80 minutes, the hemi-circumferential wall defect was left open ( Fig. 4 , Fig. 5 ), since this approach has been demonstrated to be safe after surgical local resections 1 . The patient received broad spectrum antibiotics and a liquid diet for 1 week. She was hospitalized for 2 days and had an uneventful recovery. Endoscopy 1 month later confirmed complete wound healing and the patient received local radiotherapy. At 2 years of endoscopic and radiologic follow-up, she remains asymptomatic without evidence of local recurrence or distal metastasis on imaging and endoscopy

**Fig. 2:**
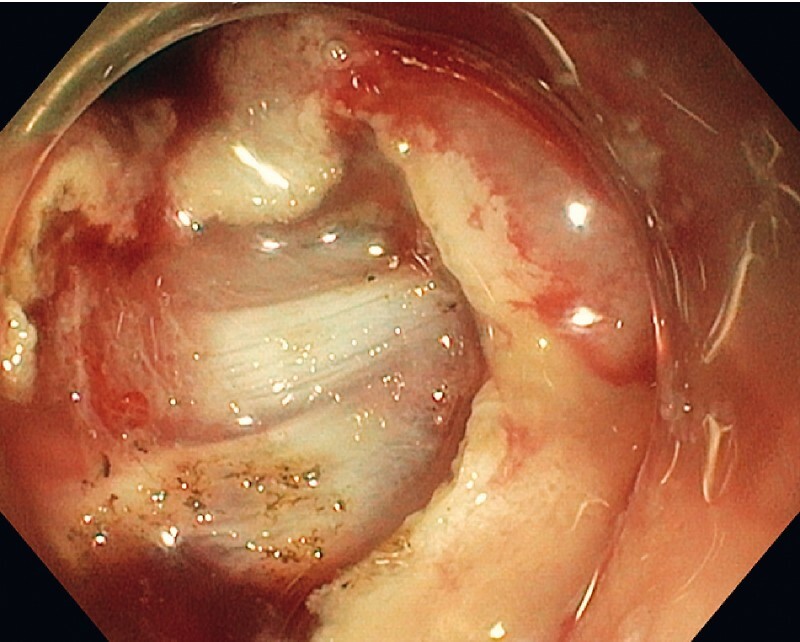
Circumferential mucosal incision.

**Fig. 3:**
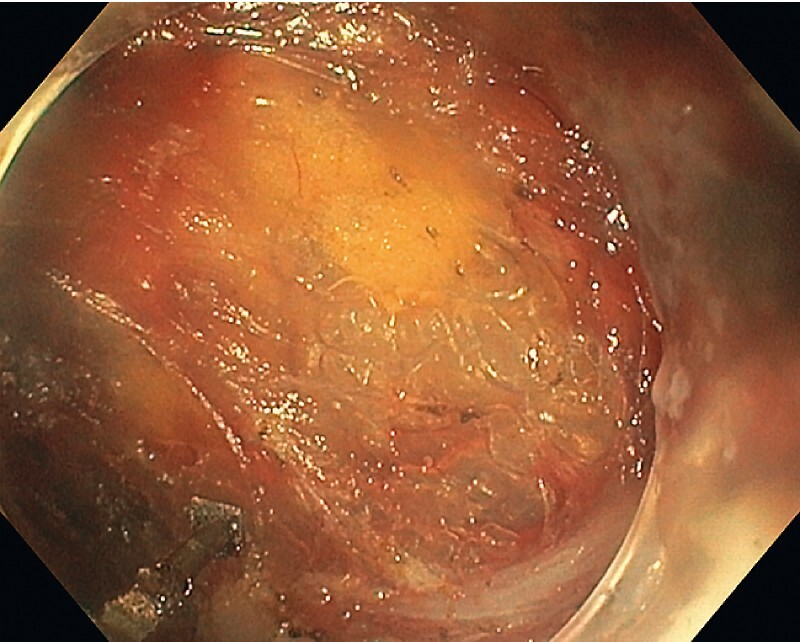
Circumferential full thickness incision.

**Fig. 4:**
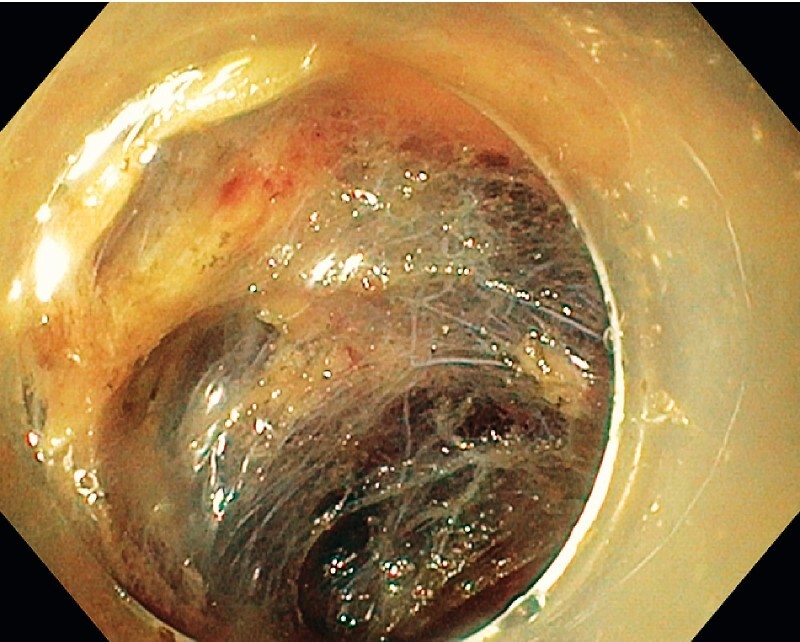
Perirectal space.

**Fig. 5:**
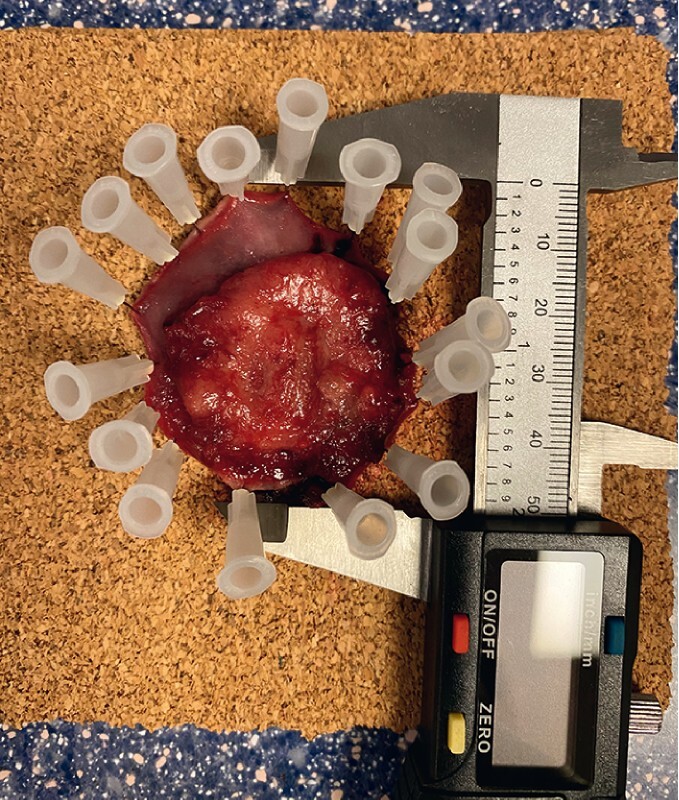
Resected specimen.

In conclusion, we presented an endoscopic salvage resection of a symptomatic T2 rectal cancer in a patient who was a poor candidate for transabdominal surgery. Local excision is an acceptable treatment for T1N0 early rectal cancer, however there is limited data for high-risk T1 and T2 tumors 2 3 . In a meta-analysis, pT1/pT2 rectal cancers treated with local excision and adjuvant (chemo)radiotherapy were associated with a 14 % local recurrence rate and 9 % distant recurrence 4 . Although this approach cannot be generalized, we demonstrated the feasibility of endoscopic excision in highly selected cases.

Endoscopy_UCTN_Code_TTT_1AQ_2AD

## Notes

Endoscopy E-Videos

https://eref.thieme.de/e-videos
*E-Videos* is an open access online section of the journal *Endoscopy* , reporting on interesting cases and new techniques in gastroenterological endoscopy. All papers include a high-quality video and are published with a Creative Commons CC-BY license. Endoscopy E-Videos qualify for HINARI discounts and waivers and eligibility is automatically checked during the submission process. We grant 100% waivers to articles whose corresponding authors are based in Group A countries and 50% waivers to those who are based in Group B countries as classified by Research4Life (see: https://www.research4life.org/access/eligibility/ ). This section has its own submission website at https://mc.manuscriptcentral.com/e-videos
